# Natural Sulfurs Inhibit LPS-Induced Inflammatory Responses through NF-κB Signaling in CCD-986Sk Skin Fibroblasts

**DOI:** 10.3390/life11050427

**Published:** 2021-05-10

**Authors:** Nipin Sp, Dong Young Kang, Hyoung Do Kim, Alexis Rugamba, Eun Seong Jo, Jong-Chan Park, Se Won Bae, Jin-Moo Lee, Kyoung-Jin Jang

**Affiliations:** 1 Department of Pathology, School of Medicine, Institute of Biomedical Science and Technology, Konkuk University, Chungju 27478, Korea; nipinsp@konkuk.ac.kr (N.S.); kdy6459@kku.ac.kr (D.Y.K.); hdkim0424@konkuk.ac.kr (H.D.K.); ra0909@konkuk.ac.kr (A.R.); 2 Pharmacological Research Division, National Institute of Food and Drug Safety Evaluation, Osong Health Technology Administration Complex, Cheongju 28159, Korea; eses0706@korea.kr (E.S.J.); elzem@korea.kr (J.-M.L.); 3 Plant Systems Engineering Research Center, Korea Research Institute of Bioscience & Biotechnology, Daejeon 34141, Korea; reredfox@kribb.re.kr; 4 Department of Chemistry and Cosmetics, Jeju National University, Jeju 63243, Korea; swbae@jejunu.ac.kr

**Keywords:** CCD-986Sk, LPS, NTS, MSM, TLR4, NF-κB, ROS, anti-inflammatory effect

## Abstract

Lipopolysaccharide (LPS)-induced inflammatory response leads to serious damage, up to and including tumorigenesis. Natural mineral sulfur, non-toxic sulfur (NTS), and methylsulfonylmethane (MSM) have anti-inflammatory activity that may inhibit LPS-induced inflammation. We hypothesized that sulfur compounds could inhibit LPS-induced inflammatory responses in CCD-986Sk skin fibroblasts. We used Western blotting and real-time PCR to analyze molecular signaling in treated and untreated cultures. We also used flow cytometry for cell surface receptor analysis, comet assays to evaluate DNA damage, and ELISA-based cytokine detection. LPS induced TLR4 activation and NF-κB signaling via canonical and protein kinase C (PKC)-dependent pathways, while NTS and MSM downregulated that response. NTS and MSM also inhibited LPS-induced nuclear accumulation and binding of NF-κB to proinflammatory cytokines COX-2, IL-1β, and IL-6. Finally, the sulfur compounds suppressed LPS-induced ROS accumulation and DNA damage in CCD-986Sk cells. These results suggest that natural sulfur compounds could be used to treat inflammation and may be useful in the development of cosmetics.

## 1. Introduction

The skin is considered the largest organ of the human body, acting as a protective barrier against environmental harms, playing a vital role in pathogen defense, and maintaining homeostasis [1]. A rupture in this system activates the immune response to maintain structural and functional integrity by inducing intercellular molecular signaling and matrix remodeling [2]. Human fibroblasts also mediate the inflammatory immune response by producing soluble signals such as growth factors, cytokines, and lipid mediators, as well as producing insoluble regulatory signals such as extracellular matrix proteins [3]. Fibroblasts facilitate wound healing and structural rearrangement by producing soluble inflammatory mediators in the immune response to infections [4,5]. Fibroblasts produce functionally active toll-like receptors (TLRs) and secrete proinflammatory cytokines such as tumor necrosis factor-α (TNFα), interferon gamma (INFγ), interleukin 6 (IL-6), IL-10, and various chemokines and growth factors in response to inflammation [6].

Lipopolysaccharide (LPS) initiates an inflammatory response in non-immune cells by stimulating the innate immune system through the pathogen-associated molecular pattern (PAMP) [7]. It neutralizes bacterial proinflammatory factors to limit the innate inflammatory response, which leads to TLR activation and is therefore used as a treatment approach in bacterial infections of the skin [8]. Studies in RAW264.7 murine macrophage cells have showed that glucosamine, an amino sugar, provides anti-inflammatory activity, inhibiting cyclooxygenase-2 (COX-2) and inducible nitric oxide (iNOS), as well as p38-MAP kinase and nuclear transcription factor NF-kappaB (NF-κB), upon stimulation with LPS [9,10].

Generally, the skin mediates innate immunity by alerting the body through systems such as PAMPs, which activate pattern recognition receptors or TLRs, thereby initiating a defense mechanism against inflammation. TLR2 and TLR4 are activated in the inflammatory response to LPS [11]. In response to pathogen invasion, TLRs recognize molecular patterns and activate the innate immune response by producing inflammatory cytokines [12,13]. TLRs also activate NF-κB signaling, which plays a vital role in inflammation by activating inflammatory genes that promote monocyte adhesion to endothelial cells [14,15]. TLRs regulate the expression of proinflammatory cytokines such as tumor necrosis factor-α (TNF-α) and members of the interleukin (IL)-1 receptor (IL-1R) family through its common adaptor myeloid differentiation factor 88 (MyD88), loss of which leads to an anti-inflammatory process [16].

In response to inflammation, TLRs activate canonical and noncanonical NF-κB pathways. In the canonical pathway, NF-κB activation occurs in response to stimuli and activates cytokine receptors in response to inflammation via the multi-subunit IκB kinase (IKK) complex and IκBα degradation [17]. The canonical pathway activated by IKKβ phosphorylation then promotes IκBα phosphorylation and ubiquitination, leading to nuclear translocation of NF-κB [18]. The IKK complex takes part in the inflammatory process through the development and differentiation of the epidermis by IKKα and NF-κB activation through IKKβ [19,20]. The noncanonical NF-κB pathway activates in response to specific stimuli and TNF receptor superfamily ligands and activates p52/RelB NF-κB complex [21]. Protein kinase C-dependent α (PKC-α) takes part in cell growth and differentiation and plays a key role in inflammation by acting as a signaling mediator between TLRs and NF-κB [22]. In inflammation, PKC-α regulates IL-1β release through ERK1/2 and p38 MAPK phosphorylation, which then activates NF-κB signaling and production of inflammatory cytokines TNF-α and IL-6 [23,24].

Several sulfur compounds are known to possess anti-inflammatory activities [25]. Sulfur is an essential element present in amino acids such as methionine and cysteine, and sulfur is consumed in the diet in foods such as duck meat, onion, or garlic [26]. In order for the toxic substances from natural mineral sulfur to be eliminated, it is typically coated with other non-toxic substances, enabling its use in the treatment of various diseases, including those associated with inflammation [27]. Methylsulfonylmethane (MSM) is a natural sulfur compound with antioxidant, anti-inflammation, anti-ketosis, and anti-cancer activities [28,29,30,31,32]. In cardiac cells, MSM inhibits inflammatory responses by regulating TNF-α signaling [30]. Non-toxic sulfur (NTS) has been used in livestock feed to enhance immunity and meat quality, and studies have shown that oral administration of NTS in rats does not induce cell death [33,34]. NTS exhibits anti-inflammatory activity against human THP-1 cells [22] and enhances growth hormone signaling in mouse C2C12 cells [35].

This study explored the activities of sulfur compounds against LPS-induced inflammation in human skin cells. We also analyzed the NF-κB-mediated molecular signaling pathway in the response to LPS and its regulation by NTS and MSM.

## 2. Material and Methods

### 2.1. Antibodies and Cell Culture Reagents

The human skin fibroblast cell line CCD-986Sk was purchased from the ATCC (CRL-1947). Iscove’s modified Dulbecco’s medium (13390), fetal bovine serum (FBS; 12003C), LPS (L2630), and MSM (1437600) were purchased from Sigma-Aldrich (Merck KGaA, St. Louis, MO). Penicillin–streptomycin solution and trypsin–EDTA (0.05%) were purchased from Gibco (Thermo Fisher Scientific, Waltham, MA). Primary antibodies specific for TLR4 (sc-293072), p-Iκ-Bα (sc-8404), COX-1 (sc-19998), COX-2 (sc-47778), β-actin (sc-47778), and secondary antibodies (anti-mouse (sc-516102) and anti-rabbit (sc-2357)) were obtained from Santa Cruz Biotechnology, Inc. (Dallas, TX). Primary antibodies specific for NF-κB (#8242), Iκ-Bα (#9242), p-IKKα/β (#9958), pERK (#9101), ERK (#9102), p-p38 (#4511), p38 (#8690), and IL-1β (#12703) were purchased from Cell Signaling Technology, Inc. (Beverly, MA). The PKC-α (ab179523), p-PKC-α (ab59411), IL-6 (ab6672), and TBP (ab818) antibodies were obtained from Abcam (Cambridge, MA). A primary antibody specific for iNOS (NB300-650) was purchased from Novus Biologicals (Littleton, CO). NTS was obtained from NaraBio (Gunsan, Korea).

### 2.2. Cell Culture and Treatment

In order to analyze the anti-inflammatory action of sulfur compounds for the development of cosmetic purposes, we used skin fibroblast and CCD-986Sk cells, maintained in Iscove’s modified Dulbecco’s medium supplemented with 10% FBS and 100 U/mL penicillin at 37 °C and 5% CO_2_. Cells were cultured (1 × 10^6^ cells/mL) for 48 h in 10 ng/mL LPS containing indicated concentrations of NTS or MSM. NTS was used in micrograms per milliliter as the molecular weight of NTS was yet to be found, and MSM was used as a molar solution (mM). Cell supernatants, lysates, and RNA were harvested for ELISA, Western blotting, real-time quantitative polymerase chain reaction (qPCR), and comet assays.

### 2.3. Cell Viability Assay

Cell viability was assayed by measuring blue formazan that was metabolized from 3-(4,5-dimethylthiazol-2-yl)-2,5-diphenyl tetrazolium bromide (MTT) by mitochondrial dehydrogenase. The cells were resuspended in the medium 1 day before treatment, at a density of 3 × 10^3^ cells per well in 96-well culture plates. CCD-986Sk cells were incubated with or without 10 ng/mL LPS and various NTS or MSM concentrations. MTT (5 mg/mL) was added to each well and incubated for 3 h at 37 °C. The formazan product was dissolved by adding DMSO, and absorbance was measured at 570 nm on an Ultra Multifunctional Microplate Reader (TECAN, Durham, NC). All measurements were performed in triplicate and were repeated at least three times.

### 2.4. Real-Time qPCR

Total RNA was extracted using an RNeasy Mini Kit (Qiagen GmbH, Hilden, Germany) according to the manufacturer’s protocol. The isolated RNA was quantified spectrophotometrically at 260 nm, and cDNA was synthesized at 42 °C for 1 h and 95 °C for 5 min with oligo d(T) primers and a first-strand cDNA synthesis kit (K-2041; Bioneer Corporation, Daejeon, Korea). Real-time qPCR was conducted in a thermal cycler (C1000 Thermal Cycler, Bio-Rad, Hercules, CA) as follows: 2 μL of diluted cDNA was added to diluted forward and reverse primers (1 μL each, 100 pM) and 8 μL TB Green Advantage Premix (Takara Bio, Japan). Cycling conditions were as follows: initial denaturation at 95 °C for 5 min, followed by 40 cycles of denaturation at 95 °C for 40 s, annealing at 58 °C for 40 s, extension at 72 °C for 40 s, and a final extension at 72 °C for 5 min. All measurements were performed in triplicate. Relative target gene expression was normalized to GAPDH. The calculations were performed using the Cp values obtained from the machine.

### 2.5. Western Blotting

Whole-cell lysates were prepared from cells treated with or without LPS and NTS or MSM by incubating them on ice with a radioimmunoprecipitation lysis buffer (20-188; EMD Millipore) containing phosphatase and protease inhibitors. Nuclear proteins were isolated using a nuclear extraction kit (ab113474). Protein concentrations were measured using the Bradford method (Thermo Fisher Scientific, Inc., Waltham, MA). Equal amounts of proteins (150 μg/well) were resolved by 10–15% SDS-PAGE. Then, the separated proteins were transferred to nitrocellulose membranes. The blots were blocked for 1 h with 5% skim milk (BD Biosciences, CA; 90002-594) in TBS-T buffer (20 mM Tris–HCl (Sigma-Aldrich; Merck KGaA, St. Louis, MO; 10708976001), pH 7.6; 137 mM NaCl (Formedium, Norfolk, UK; NAC03); 0.1 × Tween 20 (Scientific Sales, Inc. Oak Ridge, TN; 0777)) at room temperature. The membranes were then incubated with primary antibodies diluted in 5% skim milk overnight at 4 °C in a shaker. The membranes were washed with TBS-T and incubated for 1 h with horseradish peroxidase-conjugated secondary antibodies at room temperature. Detection was conducted with a Femto Clean Enhanced Chemiluminescence Solution Kit (GenDEPOT; 77449; Katy TX), and images were acquired using a LAS-4000 imaging device (Fujifilm, Tokyo, Japan).

### 2.6. Flow Cytometry Analysis for TLR4 and Cellular ROS Detection

After cultured cells were washed with pre-chilled PBS, cell pellets were incubated with 10% BSA on ice for 20 min. The following antibodies (1/200 diluted) were used for staining on ice for 30 min: PE anti-human CD282 (TLR4) (Biolegend; 312806). Stained cells were washed with pre-chilled PBS. For cellular ROS, cells were stained with CM-H2DCFDA (5 μM; Invitrogen; C6827) and placed in a CO_2_ incubator at 37 °C for 30 min. The stained cells were washed with 1 mL of prewarmed staining buffer. Cytometry analysis was performed using a FACS Calibur flow cytometer (BD Bioscience).

### 2.7. Comet Assay

The comet assay kit (ab238544) for measuring cellular DNA damage was purchased from Abcam (Cambridge, MA, USA). This assay is a single-cell gel electrophoresis method for a simple evaluation of cellular DNA damage. A base layer of comet agarose was created on a slide, and then a layer of cells treated with 10 ng/mL LPS containing the indicated concentrations of NTS or MSM was added, followed by another layer of agarose, followed by lysis. Electrophoresis was performed under neutral conditions, and the cells were stained with DNA dye. Cell morphology was observed by fluorescence microscopy (Olympus IX71/DP72).

### 2.8. ELISA

Cells were cultured (1 × 10^6^ cells/mL) for 48 h in 10 ng/mL LPS containing the indicated concentrations of NTS or MSM. IL-1β and IL-6 were measured in the supernatants of cultured cells using a human ELISA kit (Abcam; IL-1β: ab46052 and IL-6: ab178013) according to the manufacturer’s instructions.

### 2.9. Statistical Analyses

All experiments were conducted at least three times. Results are expressed as the mean ± standard error of the mean. Statistical analyses were conducted via one-way analysis of variance (ANOVA) with Tukey’s post hoc test using the SAS 9.3 software program (SAS Institute, Inc., Cary, NC). A *p*-value < 0.05 (*) was considered to indicate a statistically significant difference. # indicates the significance level.

## 3. Results

### 3.1. Sulfur Compound-Induced Cytoprotection Against LPS-Induced Cell Death and Inhibition of TLR4 Expression

**Figure 1:**
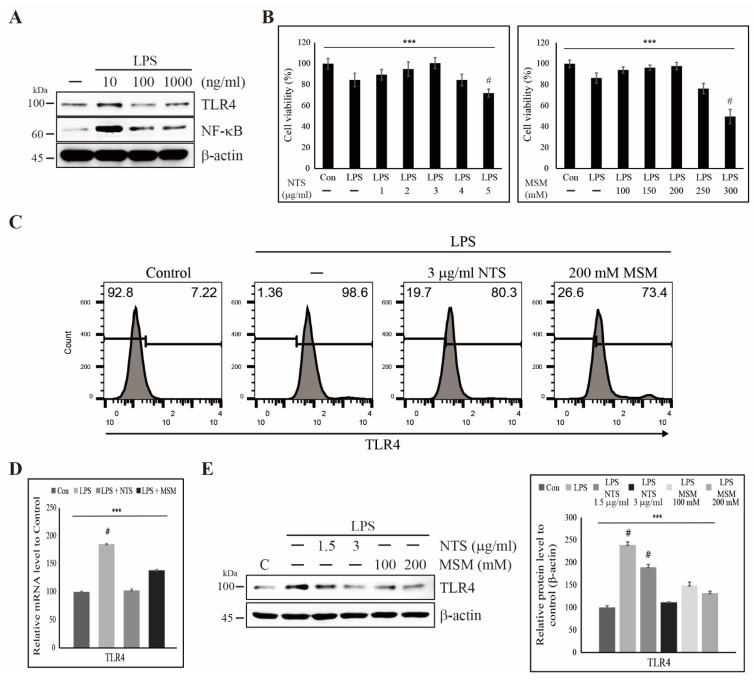
Sulfur compounds inhibited LPS-induced expression of TLR4. (**A**) Western blot analysis in CCD-986Sk cells treated with 10, 100, and 1000 ng/mL LPS for 48 h showing TLR4 and NF-κB expression. (**B**) MTT assay showing cell viability with 10 ng/mL LPS and increasing concentrations of NTS or MSM. Data are representative of three independent experiments. *** p < 0.001 (ANOVA test). # The mean difference is significant at the 0.001 level. (**C**) Flow cytometry showing inhibition of LPS-induced expression of TLR4 by 3 µg/mL NTS and 200 mM MSM for 48 h. (**D**) Real-time PCR data of mRNA after treatment with 10 ng/mL LPS and 3 µg/mL NTS or 200 mMMSM for 48 h showing the relative expression of TLR4 normalized to GAPDH. *** p < 0.001 (ANOVA test). # The mean difference is significant at the 0.001 level. (**E**) Western blotting analysis of TLR4 expression in CCD-986Sk cells treated with 10 ng/mL LPS and NTS (1.5 and 3 ng/mL) or MSM (100 and 200 mM) for 48 h. Expression levels were determined by densitometry and normalized to β-actin. Data are representative of three independent experiments. *** p < 0.001 (ANOVA test). # The mean difference is significant at the 0.001 level.

To evaluate the effect of LPS in human skin fibroblasts, CCD-986Sk, we first analyzed the dose-dependent activity of LPS. We found an increase in TLR4 and NF-κB with 10 ng/mL LPS, while higher concentrations of LPS reduced expression of TLR4 and NF-κB, perhaps being due to the induction of cell death (Figure 1A). We also analyzed cell death induced by LPS and sulfur compounds and found that the addition of LPS produced around 20% cell death, whereas lower concentrations of NTS and MSM provided cytoprotection against LPS-induced cell death. Cell viability increased with increasing NTS concentration to 3 µg/mL and MSM until 200 mM (Figure 1B). We hypothesize that this cytoprotective effect may help to inhibit inflammatory responses to LPS. Flow cytometry showed an increase in TLR4 expression in response to LPS, which was reversed by 3 µg/mL NTS or 200 mM MSM (Figure 1C). We evaluated the expression of TLR4 at the transcriptional and translational level and verified inhibition by 3 µg/mL NTS or 200 mM MSM at the transcript level (Figure 1D) and concentration-dependent inhibition of LPS-induced TLR4 protein expression (Figure 1E). These results suggest that sulfur compounds may be used for treatment against LPS-induced inflammation.

### 3.2. Suppression of LPS-Induced ROS by Sulfur Compounds in Human Skin Cells

**Figure 2:**
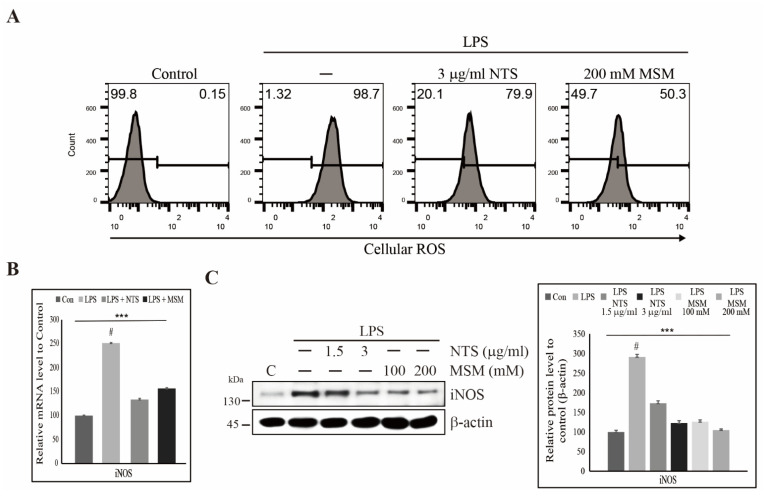
Sulfur compounds inhibited LPS-induced ROS. (**A**) Flow cytometry analysis of cellular ROS in CCD-986Sk cells after treatment with 10 ng/mL LPS and 3 µg/mL NTS or 200 mM MSM for 48 h. (**B**) Real-time PCR of iNOS mRNA after treatment with 10 ng/mL LPS and 3 µg/mL NTS or 200 mM MSM after 48 h, data normalized to GAPDH. *** p < 0.001 (ANOVA test). # The mean difference was significant at the 0.001 level. (**C**) Western blotting analysis of iNOS expression in CCD-986Sk cells treated with 10 ng/mL LPS and NTS (1.5 and 3 ng/mL) or MSM (100 and 200 mM) for 48 h. The representative expression levels of proteins following NTS or MSM treatment were determined by densitometry and normalized to β-actin. Data are representative of three independent experiments. *** p < 0.001 (ANOVA test). # The mean difference was significant at the 0.001 level.

We observed a cytoprotective effect of NTS and MSM against LPS-induced cell death. We thus assumed that these sulfur compounds might inhibit LPS-induced ROS formation. Flow cytometry of cellular ROS supported our assumption, as 3 µg/mL NTS or 200 mM downregulated LPS-induced ROS formation (Figure 2A). ROS induction occurs due to the activation of iNOS, and to confirm the induction of iNOS, we analyzed mRNA and protein levels of iNOS expression. Analysis of iNOS expression at the transcript level showed an increase in iNOS expression in response to LPS treatment, inhibited by NTS or MSM treatment (Figure 2B). Protein and transcript expression analyses confirmed the dose-dependent inhibition of LPS-induced ROS production by NTS or MSM (Figure 2C). These results provide strong support for the cytoprotective effect of sulfur compounds.

### 3.3. Sulfur Compounds Inhibited LPS-Induced DNA Damage in CCD-986Sk Cells

**Figure 3:**
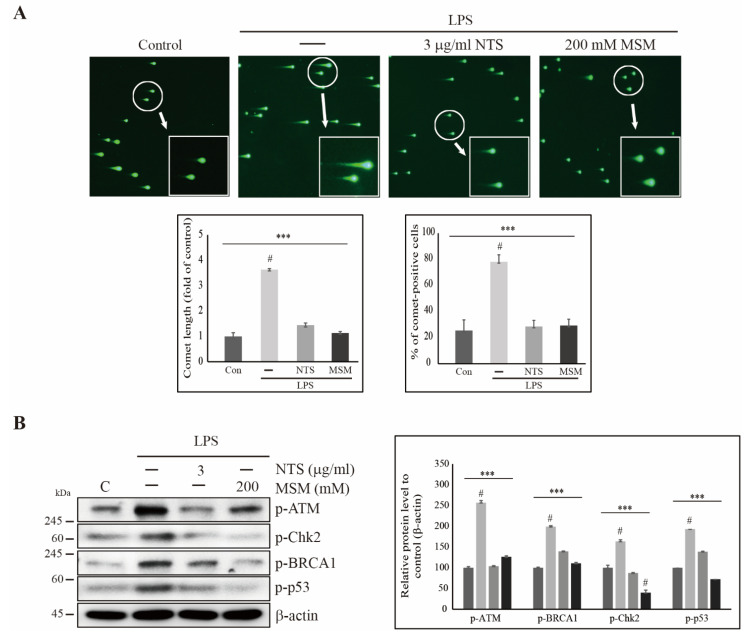
Sulfur compounds inhibited LPS-induced DNA damage. (**A**) Comet assay imaged by electron microscopy for 10× and 40× magnification showing fragmented DNA migrated out of the nucleoid body and formed a comet tail after treatment with 10 ng/mL LPS, 3 µg/mL NTS, or 200 mM MSM for 48 h. Representative comet length and comet-positive cells were analyzed as the fold change versus the control. Statistical analysis was performed using the ANOVA test (*** *p* < 0.001). # Mean difference was significant at the 0.001 level. (**B**) Western blot analysis in CCD-986Sk cells showing that 3 µg/mL of NTS or 200 mM of MSM inhibited LPS-induced expressions of p-ATM, p-Chk2, p-BRCA1, and p-p53. Expression was determined by densitometry and normalized to β-actin. The values are presented as means ± SEM of three independent experiments performed in duplicate (*n* = 3). *** *p* < 0.001 (ANOVA test). # Mean difference was significant at the 0.001 level.

Prolonged ROS induction may lead to DNA damage. Thus, we used comet assays to explore whether LPS induces DNA damage and if sulfur compounds reverse these effects. Fluorescent microscopy showed an increase in comet length and number of comet-positive cells after LPS treatment, and these effects were reversed by 3 µg/mL NTS or 200 mM (Figure 3A). We analyzed the expression of molecular signals of DNA damage response (DDR). We observed increased expression of phosphorylated ataxia-telangiectasia mutated (ATM), checkpoint kinase 2 (Chk2), breast cancer 1 (BRCA1), and tumor protein 53 (p53), all of which are markers of DNA damage in the presence of LPS (Figure 3B). NTS and MSM significantly reduced the expression of these DNA damage response signals, indicating the ability of sulfur compounds to induce DDR in LPS-induced inflammation.

### 3.4. Inhibition of LPS-Induced Canonical Pathway and PKC-Mediated Upstream Targets of NF-κB by Sulfur Compounds

**Figure 4:**
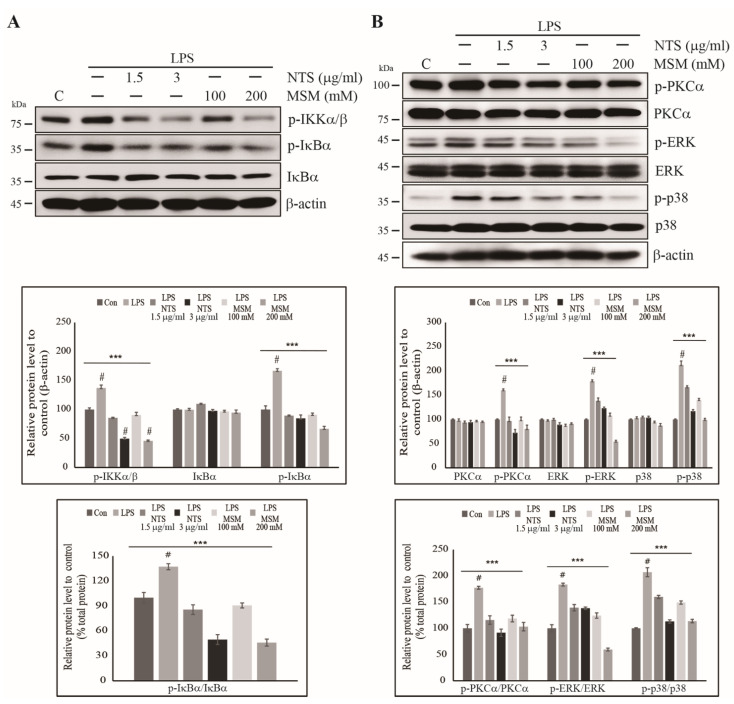
Sulfur compounds inhibited the LPS-induced canonical and PKC-dependent NF-κB pathways. (**A**) Western blotting analysis in CCD-986Sk cells treated with 1.5 and 3 µg/mL of NTS or 100 and 200 mM of MSM for 48 h showing the inhibition of 10 ng/mL LPS-induced p-IKKα/β, IκBα, and p-IκBα expression. Protein expression was determined by densitometry and normalized to β-actin. Values are presented as means ± SEM of three independent experiments performed in duplicate (*n* = 3). *** p < 0.001 (ANOVA test). Relative expressions of p-IκBα protein were determined via densitometry and normalized to total IκBα protein. *** *p* < 0.001. # Mean difference was significant at the 0.001 level. (**B**) Western blotting analysis in CCD-986Sk cells treated with 1.5 and 3 µg/mL of NTS or 100 and 200 mM of MSM for 48 h showing inhibition of 10 ng/mL LPS-induced p-PKC-α, p-ERK, and p-p38 expression along with unchanged expressions of PKC-α, ERK, and p38 proteins. Protein expression was determined by densitometry and normalized to β-actin. The values are presented as means ± SEM of three independent experiments performed in duplicate (*n* = 3). *** p < 0.001 (ANOVA test). Relative expressions of p-PKC-α, p-ERK, and p-p38 were determined via densitometry and normalized to their total PKC-α, ERK, and p38 proteins. *** *p* < 0.001. # Mean difference was significant at the 0.001 level.

We saw an increase in the expression of NF-κB in response to 10 ng/mL LPS in CCD-986Sk cells, indicating an inflammatory response. We then explored the involvement of NF-κB in this inflammatory response. First, we assessed the expression of the canonical NF-κB pathway and observed upregulation in of IKKα/β and IκBα phosphorylation, both of which showed a dose-dependent reduction in the presence of NTS of MSM (Figure 4A). Total IκBα expression remained similar in the control and treated cells. Ratio of phosphorylated IκBα protein to its total form also showed similar result to the phosphorylated proteins compared with β-actin. These results suggest that NTS and MSN inhibit the LPS-induced inflammatory response via the canonical NF-κB pathway. Then, we analyzed PKC-mediated upstream targets of NF-κB, which are also considered key members of the inflammatory response pathway. The results were similar to that of the canonical NF-κB pathway, as LPS increased expression of phosphorylated PKC-α, ERK, and p38 signaling, and this effect was reversed with NTS or MSM, with total PKC-α and ERK protein expression and p38 signaling, although the difference was non-significant. Ratio of phosphorylated PKC-α, ERK, and p38 proteins to their total form also showed similar result to the phosphorylated proteins compared with β-actin (Figure 4B). These results provide strong support for a putative mechanism of inhibition of LPS-induced inflammation by sulfur compounds via the NF-κB pathway.

### 3.5. Sulfur Compounds Inhibited LPS-Induced COX-2 and Proinflammatory Cytokines

**Figure 5:**
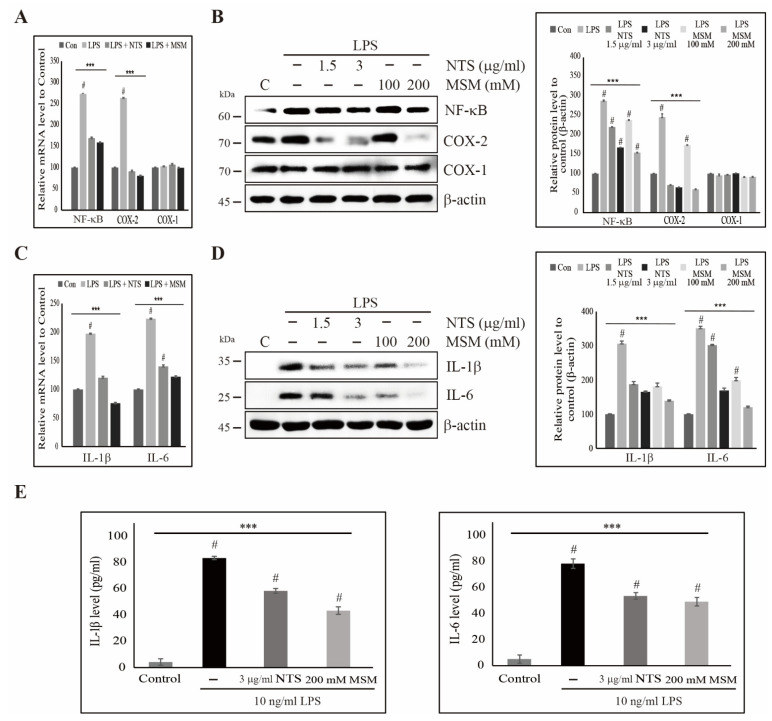
Sulfur compounds inhibited the LPS-induced NF-κB, COX-2, and proinflammatory cytokines. (**A**) Real-time PCR data of mRNA after treatment with 10 ng/mL LPS and 3 µg/mL NTS and 200 mM MSM for 48 h showing the relative expression levels of NF-κB, COX-1, and COX-2 normalized to GAPDH mRNA. *** p < 0.001 (ANOVA test). # The mean difference was significant at the 0.001 level. (**B**) Western blotting analysis in CCD-986Sk cells treated with 10 ng/mL LPS and NTS (1.5 and 3 ng/mL) or MSM (100 and 200 mM) for 48 h showing the expression of NF-κB, COX-1, and COX-2. The representative expression levels of proteins following NTS or MSM treatment were determined by densitometry and normalized to β-actin. Data are representative of three independent experiments. *** p < 0.001 (ANOVA test). # The mean difference was significant at the 0.001 level. (**C**) Real-time PCR data of mRNA after treatment with 10 ng/mL LPS and 3 µg/mL of NTS and 200 mM of MSM for 48 h showing the relative expression levels of IL-1β and IL-6 normalized to GAPDH mRNA. *** p < 0.001 (ANOVA test). # The mean difference was significant at the 0.001 level. (**D**) Western blotting analysis in CCD-986Sk cells treated with 10 ng/mL LPS and NTS (1.5 and 3 ng/mL) or MSM (100 and 200 mM) for 48 h showing the expression of IL-1β and IL-6. The representative expression levels of proteins following NTS or MSM treatment were determined by densitometry and normalized to β-actin. Data are representative of three independent experiments. *** p < 0.001 (ANOVA test). # The mean difference was significant at the 0.001 level. (**E**) ELISA analysis showing the inhibition of the LPS-induced expression of IL-1β and IL-6 by 3 µg/mL of NTS and 200 mM of MSM. Data are representative of three independent experiments. *** p < 0.001 (ANOVA test). # The mean difference was significant at the 0.001 level.

Sulfur compounds inhibited LPS-induced upstream targets and the canonical NF-κB pathway. We evaluated the expression of LPS-induced NF-κB and COX-2 and the response to treatment with sulfur compounds. Transcriptional analysis in CCD-986Sk cells showed an LPS-induced increase in the expression of NF-κB and COX-2, which was reduced by 3 µg/mL NTS or 200 mM with non-significant changes in COX-1 expression (Figure 5A). Protein analysis of NF-κB and COX-2 expression were consistent with the results of transcription analysis. Sulfur compounds inhibited LPS-induced expression of NF-κB and COX-2 with unchanged COX-1 expression (Figure 5B). These results suggested that NF-κB and its neuronal target COX-2 play a vital role in LPS-induced inflammatory responses, perhaps via the release of proinflammatory cytokines. We analyzed the expression of IL-1β and IL-6 transcripts (Figure 5C) and proteins (Figure 5D) and saw consistent upregulation in the presence of LPS, which was then downregulated by increasing concentrations of NTS or MSM. We also confirmed the release of proinflammatory cytokines IL-1β and IL-6 by ELISA and found significant inhibition of LPS-induced proinflammatory cytokines by sulfur compounds (Figure 5E). These results also suggested the inhibitory effect against LPS-induced NF-κB-dependent inflammation response by sulfur compounds.

### 3.6. Downregulation of LPS-Induced Nuclear Translocation of NF-κB by Sulfur Compounds in CCD-986Sk Cells

**Figure 6:**
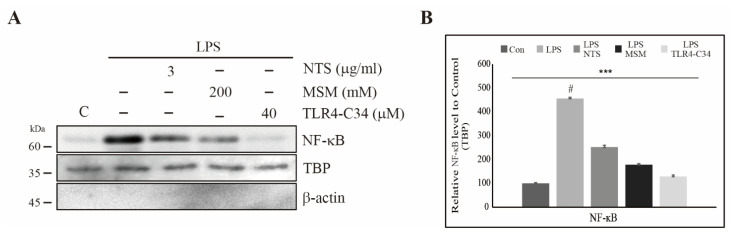
Sulfur compounds inhibited the LPS-induced nuclear accumulation of NF-κB. (**A**) Western blotting analysis of nuclear extracts harvested from CCD-986Sk cells treated with 3 µg/mL NTS or 200 mM MSM or 40 µM TLR4 inhibitor (TLR4-C34) for 48 h showing the inhibition of 10 ng/mL LPS-induced nuclear translocation of NF-κB. (**B**) Protein expression after NTS, MSM, and TLR4-C34 treatment were determined by densitometry and normalized to TBP. Data are representative of three independent experiments. *** p < 0.001 (ANOVA test). # The mean difference was significant at the 0.001 level.

**Figure 7:**
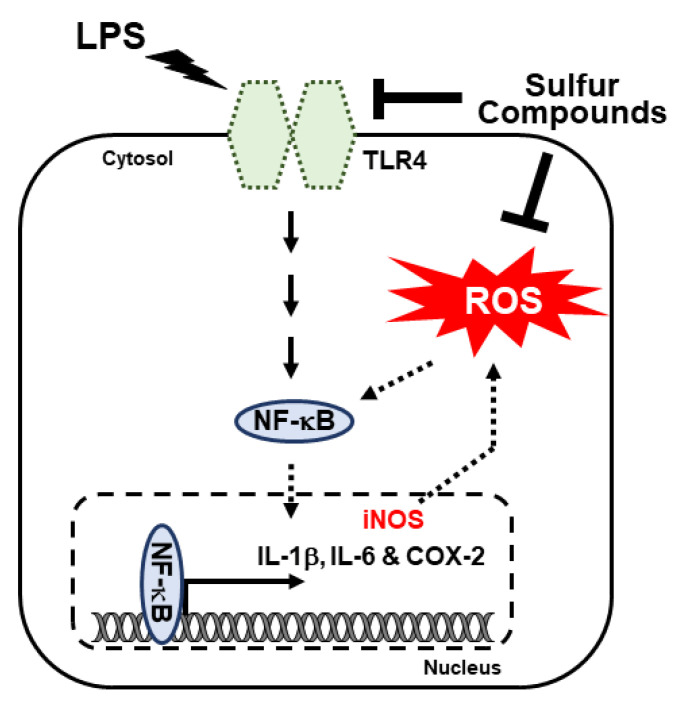
Molecular mechanism of LPS-induced regulation of the inflammatory response by TLR4/NF-κB through canonical and PKC-mediated pathways. The anti-inflammatory activities of NTS and MSM were achieved by inhibiting these pathways and binding of NF-κB to proinflammatory cytokines.

We showed that sulfur compounds inhibited LPS-induced NF-κB expression at the transcript and protein levels. We then assessed the nuclear translocation of NF-κB. Here, we extracted nuclear protein from CCD-986Sk cells treated with LPS and NTS, MSM, and a TLR4 inhibitor (TLR4-C34) and evaluated the products by Western blotting. The results showed elevated expression of NF-κB after LPS treatment, which indicated that LPS promotes nuclear translocation of NF-κB as a response to inflammation (Figure 6A). The addition of sulfur compounds or TLR4-C34 produced a significant decrease in LPS-induced translocation of NF-κB and no change in TBP expression (Figure 6B). These results also suggest that sulfur compounds can inhibit LPS-induced inflammatory responses in CCD-986Sk cells. Simply put, molecular signaling includes the inhibition of LPS-induced TLR4, the canonical and PKC-dependent NF-κB, COX2 expression, and finally inhibition of IL-1β IL-6 release (Figure 7).

## 4. Discussion

Inflammation due to LPS may lead to activation of several proinflammatory cytokines that may result in disease pathogenesis, including tumorigenesis. Chronic inflammation leads to DNA damage and subsequent tumor development due to the presence of inflammatory cells in the tumor microenvironment [36]. Natural minerals provide a useful counter to the inflammatory response as they are generally free of side effects. Sulfur-containing compounds from natural products also exhibit anti-inflammatory properties against LPS-induced inflammation [37]. NTS is a form of non-toxic mineral sulfur. Studies have shown the ability of NTS to enhance growth hormone signaling via the JAK2/STAT5b/IGF-1 axis and to produce anti-inflammatory effects against hyperglycemia in human monocytes [22,35]. MSM is another sulfur-containing natural compound with various anti-inflammatory activities [38] and exercise-induced stress in racing horse muscle cells via p53-mediated SDHA/HPRT1 expression [39]. MSM has been already tested in normal cells that show no cytotoxicity. Study of MSM in an animal model also showed no toxicity, remaining healthy and vigorous to a concentration of 6–8 g/kg/day through oral administration [40]. In our previous studies on toxicity analysis of MSM in human monocytes, THP-1 cells suggested very low or negligible cytotoxicity in THP-1 cells by 200 mM MSM [22]. We also studied cytotoxic effects of MSM in bone marrow-derived macrophages isolated from mice, finding no toxicity up to 200 mM MSM [41]. We hypothesized that these sulfur compounds could inhibit LPS-induced inflammatory responses in CCD-986Sk skin fibroblasts.

TLR4 is a transmembrane receptor that could activate inflammatory signals when a pathogen attacks. LPS treatment has also been shown to activate TLR4 as an inflammatory response [42,43]. We also found similar upregulation of TLR4 caused by LPS in CCD-986Sk cells. NTS and MSM inhibited LPS-induced TLR4 expression, which suggested the anti-inflammatory activity of these sulfur compounds. We analyzed the downstream targets of TLR4 and the mechanisms underlying the anti-inflammatory activity of sulfur compounds.

The addition of LPS causes oxidative damage via ROS production, which results in tumor progression through TLR4 activation [44]. We observed a large increase in cellular ROS due to LPS, similar to TLR4 activation. We thus confirmed the induction of inflammation by LPS. As a response to inflammation due to LPS, iNOS generate NO and ROS generated by NOX2 [45]. Hence, we analysed the the ability of sulfur compounds in the regulation of key factor iNOS and ROS production. NTS or MSM downregulated LPS-induced ROS formation and iNOS expression, which suggests sulfur compounds inhibit LPS-induced inflammation. These sulfur compounds also inhibited DNA damage caused by LPS and induced DDR. DNA damage is also considered a byproduct of LPS-induced inflammation that results in tumorigenesis through DNA double-strand breaks [46]. Our results indicated that LPS induces ROS production and DNA damage due to inflammatory response signaling that was successfully suppressed and reversed by sulfur compounds via inhibition of phosphorylated ATM, Chk2, BRCA1, and p53. These findings suggest the potential utility of sulfur compounds as candidate treatments for inflammation. NF-κB also plays a key role in DNA damage process, whereas lack of study on the relationship between DNA damage and NF-κB is considered a limitation of this study. 

The LPS-induced inflammatory response stimulates immune cell activation of TLR4 and signals to its downstream targets, primarily NF-κB, the major downstream regulator of TLR4-dependent inflammatory response signaling [47,48]. During the inflammatory response, activation of NF-κB occurs through the canonical [49] and PKC-dependent pathways [50]. LPS activated both NF-κB inflammatory pathways in CCD-986Sk cells. In the canonical pathway, LPS induced expression of phosphorylated IKKα/β and IκBα; in the PKC-dependent pathway, LPS activated phosphorylation of PKC-α, ERK, and p38. NTS and MSM downregulated these effects via inhibition of TLR4/NF-κB signaling. The addition of cholesterol sulfate could be a key towards the anti-inflammatory responses of sulfur compounds as cholesterol sulfate attenuates the inflammatory responses by regulating NF-κB and PPARγ signaling [51]. 

NF-κB is an inflammatory transcriptional regulator of the genes encoding proinflammatory cytokines such as COX-2, TNF-α, IL-1β, and IL-6 [52]. NF-κB controls the activity of the COX-2 gene through nuclear accumulation and IκB kinase and thereby regulates the expression of prostaglandin E2 (PGE-2), and NF-κB also controls the expression of NO through iNOS signaling [53]. LPS induces the expression of NF-κB, COX-2, and other proinflammatory cytokines such as IL-1β and IL-6, and this effect was inhibited by NTS and MSM. These results suggest that sulfur compounds reduce LPS-induced inflammation via TLR4/NF-κB regulation and subsequent inhibition of the proinflammatory cytokines COX-2, IL-1β, and IL-6. Nuclear protein expression validated this finding, showing that sulfur compounds inhibited LPS-induced expression of NF-κB via IκB kinase, which blocked the nuclear accumulation of NF-κB. Our results support the model of NTS and MSN anti-inflammatory activity by inhibiting LPS-induced expression of TLR4, NF-κB, and proinflammatory cytokines. Eventhough, our experimentation with in vitro studies suggested the anti-inflammatory effects of sulfur compounds against LPS-induced inflammation in CCD-986Sk cells, in vivo study is very important to confirm these result to consider sulfur compounds as potntial candidate drugs to treat against LPS-induced inflammation condition. Absence of an animal model could be considered as a limitation of this study. 

## 5. Conclusions

In summary, NTS and MSM can inhibit the LPS-induced inflammatory response in CCD-986Sk human skin fibroblasts. The anti-inflammatory activity of these sulfur compounds includes inhibition of LPS-induced expression of TLR4 and the canonical and PKC-dependent NF-κB pathway via the proinflammatory cytokines COX-2, IL-1β, and IL-6. NTS and MSM also suppressed LPS-induced ROS formation and DNA damage in CCD-986Sk fibroblasts. Our results suggest these natural sulfur compounds may serve as candidate therapies in LPS-dependent inflammation and may have particular utility in cosmetics production.

## Data Availability

The data presented in this study are available on request from the corresponding author. The data are not publicly available due to privacy.
